# Role of Socioeconomic Status in Hypertension among Chinese Middle-Aged and Elderly Individuals

**DOI:** 10.1155/2019/6956023

**Published:** 2019-10-13

**Authors:** Xinyi Wu, Zhonghua Wang

**Affiliations:** Department of Health Economics & Management, School of Health Policy & Management, Nanjing Medical University, Nanjing 211100, China

## Abstract

Hypertension is an important global health concern. The relationship between hypertension and socioeconomic status (SES) has been extensively studied. However, the role of SES in hypertension is still controversial, and this kind of study is sorely lacking among Chinese middle-aged and elderly individuals. The data of this study come from the China Health and Retirement Longitudinal Survey (CHARLS) released in May 2017. A total of 21,126 people from all around China, with ages older than 45 years, were enrolled in the questionnaire survey. Hypertension was determined according to the entry in CHARLS (“Do you have doctor-diagnosed hypertension?”), and 17,676 people responded to this entry. The basic demographic and SES information were collected. Multivariate logistic regression was used to evaluate the risk factors of hypertension. Concentration index was used to measure inequality of hypertension incidence. Among the investigated middle-aged and elderly individuals, 5,177/17,676 (29.3%) had doctor-diagnosed hypertension. Multivariate logistic regression implied that individuals older than 55 years (OR 1.436, 95% CI 1.085–1.900 for age interval of 55–64 years; OR 2.032, 95% CI 1.455–2.839 for age interval of 65–74 years; OR 1.672, 95% CI 1.031–2.714 for age interval of older than 75 years), male (OR 0.038, 95% CI 0.595–0.986), overweight (OR 2.47, 95% CI 1.462–4.183), and diabetes (OR 3.159, 95% CI 2.129–4.687) were associated with hypertension. For society support, individuals in the lowest quintile were more likely to suffer hypertension. Concentration index results suggested that different income groups did not show inequality on hypertension incidence. Elder age, male, overweight, diabetes, and poor society support were suggested to be associated with hypertension incidence among middle-aged and elderly individuals in China. Our study provides implications for controlling and managing hypertension.

## 1. Introduction

Hypertension is a big issue of global health and poses a great challenge for public health worldwide. It has been recognized as an important risk factor for cardiovascular diseases (CVD) [1, 2]. Globally, hypertension attributes to about 50% CVD mortality. Effective control of blood pressure is valuable for preventing the incidence of CVD [3].

In China, the morbidity of hypertension had been apparently rising in the past semicentury. Since 1958, four nation-wide investigations were conducted in 1958, 1979, 1991, and 2002, and the results showed that the hypertension prevalence rate was rising from 5–11% to 24–27% [4]. It was estimated in 2010 that approximately 200 million people suffered from hypertension in China [5].

In the Chinese guidelines for the management of hypertension (version 2010) [5], which is also a guideline for hypertension diagnosis used by Chinese doctors, hypertension was defined as follows: in the absence of antihypertensive drugs, blood pressure is measured at least 3 times in different days, with a systolic blood pressure ≥140 mmHg and/or diastolic blood pressure ≥90 mmHg. Individuals who had been diagnosed as having hypertension by a doctor or used antihypertensive drugs are also defined as hypertension. In 2017, at the annual meeting of the American Heart Association (AHA), a new guideline for hypertension was proposed, redefining hypertension as blood pressure ≥130/80 mmHg [6]. However, it was also noted that this document was for guidance only, and the decision made by the clinician according to the specific situation of the patient is the most important. It reflects that hypertension is getting more and more attention and highly valued [6].

As a special part of the population, health care of middle-aged and elderly individuals always occupies an important status in the public health services system [7, 8]. Hypertension and the consequent CVD in middle-aged and elderly individuals bring a huge burden for public health [1]. Moreover, the aging problem in China is getting more and more serious. It was reported that, in 2010, there were 111 million people aged 65+ years and 19.3 million aged 80+ years, and it was estimated that the number will increase to 400 and 150 million, respectively, by 2050 [9, 10]. In the near future, China will be one of the countries with most serious aging problem [10].

It had been indicated in many researches that socioeconomic status (SES) was closely related to hypertension. However, different researches presented some conflicting results [11–14]. There are still very limited literatures discussing the relationship between SES and hypertension prevalence in low- and middle-income countries, especially China [4, 11]. China Health and Retirement Longitudinal Study (CHARLS) is a national representative longitudinal survey of the middle-aged and elderly individuals in China. Items in this survey include health circumstance, and social and economic status of persons from across the country, both urban and rural regions, aging 45 years or older [4, 11]. CHARLS provides high-quality database for studying the relationship between SES and hypertension among middle-aged and elderly individuals in China.

## 2. Methods

### 2.1. Data Source

The data used for this study came from the CHARLS database [11]. CHARLS is hosted by the National School of Development of Peking University, and performed by the Institute of Social Science Survey and Youth league committee of Peking University. The aim of the survey was to collect high-quality microdata of Chinese families and individuals older than 45 years. The results can be used to analyze the aging problem of China, promote the interdisciplinary research of the aging problem, and provide scientific basis to formulate and improve relevant policies in China. The national baseline survey of CHARLS started in 2011 and was tracked every two years. One year after the survey ended, the data were freely available to the academic community. CHARLS was performed in 2011, 2013, 2104 (“Life History Survey” special project), and 2015, covering 23 thousand interviewees of 12.2 billion families from both urban and rural regions in 28 provinces/municipalities/autonomous regions by 2015. Items in the CHARLS questionnaire mainly include basic personal information, family structure, health status, physical measurement, medical service utilization, medical insurance, assets, basic community conditions, etc. The data in this study were followed up in 2015 and released publicly in May 2017.

### 2.2. Definitions

Hypertension was determined according to the entry in CHARLS (“Do you have doctor-diagnosed hypertension?”). Doctors diagnosed hypertension based on the Chinese guidelines for the management of hypertension (version 2010) [5], in which hypertension is defined as follows: in the absence of antihypertensive drugs, blood pressure is measured at least 3 times on different days, with a systolic blood pressure ≥140 mmHg and/or diastolic blood pressure ≥90 mmHg. Individuals who had been diagnosed as having hypertension by a doctor or used antihypertensive drugs are also defined as hypertension.

Socioeconomic status (SES) is a comprehensive indicator measuring the social status of individuals and can reflect the level of social and economic status of members in the society. Income, education, and occupation, which represent different aspects of social and economic status, are regarded as three major indicators of SES [14, 15].

Concentration index (CI) is a health equity index in health economics and is used to measure health inequities associated with SES. The CI value is between −1 and 1. A positive value of CI indicates that the prevalence of hypertension tends to be among the high-income people, and a larger value indicates a greater inclination to high-income people, and vice versa for a negative CI value. A CI value of 0 indicates that people in different income groups are equally likely to have hypertension [16].

### 2.3. Research Variables

The outcome variable is hypertension, which is defined as described earlier. SES factors are divided into four aspects: demographic factors, economic factors, health and health behavior, and psychosocial factors ([Supplementary-material supplementary-material-1]). The demographic factors include gender, age (divided into four degrees: 45–54, 55–64, 65–74, and older than 75 years) and residence (urban or rural). The economic factors include educational level, income (averaged into 5 levels), working status, and medical insurance. Health and health behavior include BMI index, exercise intensity, smoking, and diabetes. BMI value was calculated by dividing weight in kilograms by height in meters squared, and the classification criteria are as follows: underweight, <18.5; normal, 18.5–23.9; and overweight, ≥24. The psychosocial factors include depression evaluation, social support (averaged into 5 levels), and social participation (whether participating in social activities).

### 2.4. Statistical Analysis

IBM SPSS Statistics 21.0 (IBM, Chicago, IL, USA) was used for statistical analysis. Multiple imputation was used to deal with the missing data. We selected factors that were significantly associated with hypertension in the univariate analysis (*P* < 0.20). Candidate variables were entered into stepwise multivariate logistic regression using a backward selection model. To better assess the association between various SES factors and hypertension prevalence, bootstrapping (10,000 resampling) analysis was used to estimate the odds ratios (OR) and 95% confidence intervals (CI). *P* < 0.05 was considered to be statistically significant. The inequity analysis of hypertension prevalence was evaluated by using the concentration index.

## 3. Results

A total of 21,126 Chinese middle-aged and elderly (45 years or older) individuals were enrolled into the questionnaire survey of CHARLS. The characteristics of demographic factors, economic factors, health and health behavior, and psychosocial factors of these people are summarized in Table 1. Among these people, 17,676 responded to the question about hypertension, and 5,177/17,676 (29.3%) had doctor-diagnosed hypertension.

The results of evaluation of the association between various SES factors and hypertension prevalence using multivariate logistic regression are shown in Table 2. Several factors were found likely to be risk factors of hypertension. Males were more likely to suffer hypertension than females based on the CHARLS results (*P*=0.038, OR 0.038, 95% CI 0.595–0.986). Age is another important factor associated with hypertension prevalence. Compared with the youngest group of 45–54 years, the other three age groups are significantly more likely to have hypertension (*P* < 0.05), especially the 65–74 years old group, with an OR value of 2.032 and 95% CI value of 1.455–2.839. The 55–64 and older than 75 years groups presented OR values of 1.436 and 1.672, and 95% CI values of 1.085–1.900 and 1.031–2.714, respectively. Besides, consistent with most other studies investigating risk factors of hypertension and CVD, overweight and diabetes were significantly associated with hypertension (*P* < 0.05); the OR values were as high as 2.473 and 3.159, with 95% CI values of 1.462–4.183 and 2.129–4.687, respectively. Moreover, social support was also found to be associated with hypertension prevalence. People with better social support tended to stay away from hypertension. Compared with individuals receiving lowest social support, people with lower support (OR 0.618, 95% CI 0.449–0.851), medium support (OR 0.625, 95% CI 0.417–0.936), and highest support (OR 0.608, 95% CI 0.432–0.856) all showed significantly lower hypertension prevalence (*P* < 0.05). We did not find other SES factors that were associated with the prevalence of hypertension in these Chinese individuals.

At last, concentration index was used to evaluate the equity of the prevalence of hypertension in different income groups. The results showed that individuals in all income groups, no matter whether men or women, were equally likely to suffer hypertension in China (Table 3).

## 4. Discussion

Hypertension poses a sever challenge to public health and had been regarded as a potential risk for CVD [1, 3]. To better control the prevalence of hypertension, investigating associated risk factors is of great significance. It had been implied in many researches that some aspects of SES are closely associated with hypertension prevalence. However, some results of this issue are controversial, and more studies need to be conducted in low- and middle-income countries, especially China [11–15, 17].

The most recent nation-wide survey on hypertension prevalence in China was conducted in 2002, and the prevalence rate was 24–27% [4]. In this study, we found that 5,177/17,676 (29.3%) people had doctor-diagnosed hypertension, which was similar to that of the 2002 survey. However, we must note that the determination of hypertension in this study was based on the result of the self-report questionnaire, and there may be a part of individuals who were unaware of their hypertension status. In was believed that the rule of halves usually applies to hypertension prevalence in low- and middle-income countries [17]. It means that half of the hypertension population are aware of their high blood pressure, half of those aware are treated, and half of those treated effectively control the blood pressure. According to this rule, about 60% of the investigated population may suffer hypertension. However, we should also point out some special conditions to put this result in perspective. With the development of economy and people's living level, more and more people are paying more attention to their body health; periodic physical examination, including blood pressure measurement, is common in Chinese middle-aged and elderly individuals. This must greatly improve the awareness of hypertension. In addition, in this study, 76.5% people come from rural regions and 43.5% people are illiterate; this relatively low SES level may also result in increased prevalence of hypertension.

Many demographic and SES factors had been associated with hypertension prevalence. Gender is a usually mentioned influence factor in hypertension. For China, the results of a systematic review in 2014 and a national survey in 2002 both indicated that the prevalence was higher among men than women [4, 17]. In addition, another research in Chongqing Province of China investigating the prevalence of pre-hypertension also implied a similar result [18]. Our result was consistent with the above, also suggesting that men are more likely to suffer hypertension. Moreover, older age, overweight, and diabetes, which are widely recognized as risk factors to hypertension [19–22], were also found to be associated with hypertension of middle-aged and elderly individuals in China. Among these, overweight and diabetes presented relatively high OR and 95% CI values, indicating their important roles in the incidence of hypertension among middle-aged and elderly individuals. Considering that the problem of metabolic diseases including obesity and diabetes is increasingly serious in China [23], we should strengthen the emphasis on this issue.

For other SES factors we investigated, poor social support was found to be associated with the incidence of hypertension. In developed countries, lower SES was believed to be associated with higher hypertension prevalence, whereas in undeveloped or developing countries, it was often observed that higher SES was associated with higher blood pressure, and this is usually explained by the opinion that in these countries, people with higher SES are also characterized with higher prevalence of obesity, and higher salt and alcohol intakes [13]. At the same time, other researches in developing countries found the same relationship between SES and hypertension prevalence [12]. Our results support the latter opinion, indicating that better SES means lower blood pressure. People with better SES take a new look at their lifestyle and dietary habit, which may explain the phenomenon. In other researches, some other SES factors were also implied to be associated with hypertension. For example, education level, income, and depression had been suggested to be related to hypertension [19, 24, 25], but negative results were found in this study. Moreover, the calculation of concentration index in different income groups in Table 3 also supports this result, which showed that individuals in different income groups were equally likely to suffer hypertension. Further investigation is needed to explore the role of other SES factors in the prevalence of hypertension, and whether specific country or race may influence the results.

## 5. Conclusions

In summary, in this study, we found that older age, male, overweight, diabetes, and poor society support were associated with the prevalence of hypertension among middle-aged and elderly individuals in China. Our study provides implications for better control and management of hypertension.

## Figures and Tables

**Table 1:** Characteristics of the investigated population.

Item	Frequency (*n* (%))
Demographic factors	
Gender	
Male	10148 (48.0)
Female	10978 (52.0)
Age (years)	
45∼	4410 (29.3)
55∼	5874 (39.0)
65∼	3209 (21.3)
75∼	1553 (10.3)
Residence	
Urban	4353 (23.5)
Rural	14189 (76.5)

Economic factors	
Education level	
Illiteracy	9178 (43.5)
Primary school	4568 (21.7)
Junior high school	4474 (21.2)
Senior high school	2343 (11.1)
University or higher	521 (2.5)
Income	
Lowest	2153 (20.1)
Lower	2140 (19.9)
Medium	2153 (20.1)
Higher	2141 (19.9)
Highest	2145 (20.0)
Working status	
Working	11847 (65.5)
Retirement	5915 (32.7)
Unemployment	312 (1.8)
Medical insurance	
No	861 (4.7)
Yes	17414 (95.3)

Health and health behavior	
BMI	
Underweight	749 (5.8)
Normal	4915 (37.9)
Overweight	7300 (56.3)
Diabetes	
No	16154 (92.2)
Yes	1376 (7.8)
Exercise scoring	
Low intensity	2955 (49.6)
Medium	991 (16.6)
High intensity	2013 (33.8)
Smoking	
No	11969 (80.1)
Yes	2968 (19.9)

Psychosocial factors	
Depression	
No	12088 (73.0)
Yes	4473 (21.2)
Social support	
Lowest	2898 (20.1)
Lower	4113 (28.5)
Medium	1763 (12.2)
Higher	2782 (19.3)
Highest	2888 (20.0)
Social participation	
No	7852 (37.2)
Yes	8967 (42.4)
Hypertension	
No	12499 (70.7)
Yes	5177 (29.3)

**Table 2:** Logistic regression analyses of factors affecting hypertension prevalence.

Factors	OR^*∗*^	95% CI^*∗*^	*P* value^*∗*^
Age (control, 45∼) (years)			
55∼	**1.436**	**1.085–1.900**	**0.011**
65∼	**2.032**	**1.455–2.839**	**0.000**
75∼	**1.672**	**1.031–2.714**	**0.037**
Gender (control, male)	**0.765**	**0.595–0.986**	**0.038**
Residence (control, urban)	0.851	0.609–1.190	0.345
Education level (control, illiteracy)			
Primary school	0.911	0.685–1.213	0.525
Junior high school	0.745	0.543–1.022	0.068
Senior high school	0.701	0.442–1.112	0.131
University or higher	0.760	0.292–1.982	0.575
Income (control, lowest)			
Lower	1.264	0.912–1.750	0.159
Medium	0.926	0.660–1.299	0.656
Higher	0.759	0.530–1.086	0.131
Highest	1.039	0.709–1.524	0.844
Working status (control, working)			
Retirement	0.516	0.260–1.025	0.059
Unemployment	0.667	0.334–1.334	0.252
Medical insurance (control, no)			
	0.771	0.446–1.331	0.351
BMI (control, normal)			
Underweight	0.960	0.563–1.638	0.882
Overweight	**2.473**	**1.462–4.183**	**0.001**
Diabetes (control, no)			
	**3.159**	**2.129–4.687**	**0.000**
Exercise scoring (control, low intensity)			
Medium	0.775	0.568–1.057	0.107
High intensity	0.810	0.634–1.037	0.094
Smoking (control, no)	0.810	0.579–1.133	0.218
Depression (control, no)	1.231	0.971–1.561	0.086
Social support (control, lowest)			
Lower	**0.618**	**0.449–0.851**	**0.003**
Medium	**0.625**	**0.417–0.936**	**0.023**
Higher	0.733	0.516–1.041	0.082
Highest	**0.608**	**0.432–0.856**	**0.004**
Social participation (control, no)			
	1.191	0.955–1.486	0.120

^*∗*^Significant differences are in bold.

**Table 3:** Inequity analysis of hypertension prevalence by concentration index.

Income group	Total		Men		Women		*P* value^*∗*^
	Mean	SD	Mean	SD	Mean	SD	
Lowest group	0.28	0.448	0.25	0.436	0.30	0.457	0.722
Lower group	0.32	0.467	0.29	0.455	0.35	0.477	**0.030**
Medium group	0.29	0.454	0.27	0.446	0.31	0.461	**0.006**
Higher group	0.29	0.456	0.28	0.448	0.31	0.462	0.083
Highest group	0.31	0.462	0.31	0.463	0.31	0.462	0.119
Total	0.30	0.458	0.28	0.450	0.31	0.464	0.865
CI	0.0102		0.0283		−0.0037		

^*∗*^Significant differences are in bold.

## Data Availability

The datasets generated during and/or analyzed during the current study are available from the corresponding author on reasonable request.

## Supplementary Materials

Supplementary Materials Supplementary Table S1: SES factors.
