# Supplementary material for: Role of Socioeconomic Status in Hypertension among Chinese Middle-Aged and Elderly Individuals
Source: Int J Hypertens. 2019 Oct 13;2019:6956023. doi: 10.1155/2019/6956023 (PMC6815568; doi:10.1155/2019/6956023)
Supplement: Supplementary Materials — Supplementary Table S1: SES factors. [file 6956023.f1.pdf]

Supplementary Table S1 SES factors

Variables	Assignment
Dependent variable	
Hypertension	0=No， 1=Yes
Independent variable	
**Demographic factors**	
Sex	0=Male, 1=Female
Age	45~, 55~, 65~, 75~
Residence	0=Urban， 1=Rural
**Economic factors**	
Education level	0= Illiteracy, 1= Primary school, 2=Junior high school, 3= Senior high school, 4=University or higher
Income	1=Lowest
	2=Lower
	3=Medium
	4=Higher
	5=Highest
Working status	0=Working, 1=Retirement, 2= Unemployment
Medical insurance	0=No, 1=Yes
**Health and health behavior**	
BMI	0= Underweight (<18.5), 1=Normal (18.5~23.9),
	2= Overweight (≥24)
Exercise	0=Low intensity, 1=Medium, 2= High intensity
Smoking	0=No, 1=Yes
Diabetes	0=No, 1=Yes
**Psychosocial factors**	
Depression	0=No, 1=Yes
Social support	1=Lowest
	2=Lower
	3=Medium
	4=Higher
	5=Highest
Social participation	0=No, 1=Yes
